# Multiple essential roles for primary cilia in heart development

**DOI:** 10.1186/2046-2530-1-23

**Published:** 2012-12-11

**Authors:** Marc August Willaredt, Karin Gorgas, Humphrey A R Gardner, Kerry L Tucker

**Affiliations:** 1 Interdisciplinary Center for Neurosciences, University of Heidelberg, Heidelberg, 69120, Germany; 2 Institute of Anatomy and Cell Biology, University of Heidelberg, Im Neuenheimer Feld 307, Heidelberg, 69120, Germany; 3 AstraZeneca, 35 Gatehouse Drive, Waltham, Massachusetts, 02451, U.S.A

**Keywords:** Primary cilia, Heart, Outflow tract, Aorta, Pulmonary trunk, Endocardial cushions, AVSD, Nkx2.5, Pitx2c, Isl1, Hand1, Alpha-actinin, Bmp2, Bmp4, Shh, Cardiac neural crest

## Abstract

**Background:**

The primary cilium is a microtubule-based, plasma membrane-ensheathed protrusion projecting from the basal bodies of almost all cell types in the mammalian body. In the past several years a plethora of papers has indicated a crucial role for primary cilia in the development of a wide variety of organs. We have investigated heart development in *cobblestone*, a hypomorphic allele of the gene encoding the intraflagellar transport protein Ift88, and uncovered a number of the most common congenital heart defects seen in newborn humans.

**Methods:**

We generated serial sections of mutant *cobblestone* and wild type embryos in the region encompassing the heart and the cardiac outflow tract. The sections were further processed to generate three-dimensional reconstructions of these structures, and immunofluorescence confocal microscopy, transmission electron microscopy, and *in situ* hybridization were used to examine signal transduction pathways in the relevant areas. Whole mount *in situ* hybridization was also employed for certain developmental markers.

**Results:**

In addition to an enlarged pericardium and failure of both ventricular and atrial septum formation, the *cobblestone* mutants displayed manifold defects in outflow tract formation, including persistent truncus arteriosus, an overriding aorta, and abnormal transformation of the aortic arches. To discern the basis of these anomalies we examined both the maintenance of primary cilia as well as endogenous and migratory embryonic cell populations that contribute to the outflow tract and atrioventricular septa. The colonization of the embryonic heart by cardiac neural crest occurred normally in the *cobblestone* mutant, as did the expression of *Sonic hedgehog*. However, with the loss of primary cilia in the mutant hearts, there was a loss of both downstream Sonic hedgehog signaling and of Islet 1 expression in the second heart field, a derivative of the pharyngeal mesoderm. In addition, defects were recorded in development of atrial laterality and ventricular myocardiogenesis. Finally, we observed a reduction in expression of *Bmp4* in the outflow tract, and complete loss of expression of both *Bmp2* and *Bmp4* in the atrioventricular endocardial cushions. Loss of BMP2/4 signaling may result in the observed proliferative defect in the endocardial cushions, which give rise to both the atrioventricular septa as well as to the septation of the outflow tract.

**Conclusions:**

Taken together, our results potentially identify a novel link between Sonic hedgehog signaling at the primary cilium and BMP-dependent effects upon cardiogenesis. Our data further point to a potential linkage of atrioventricular septal defects, the most common congenital heart defects, to genes of the transport machinery or basal body of the cilia.

## Background

In the mouse cardiogenesis begins at embryonic day 7.5 (E7.5) by migration of the anterior lateral plate mesoderm towards the midline, where it forms a linear heart tube. This primary heart field gives rise to both the left and right atria and the left ventricle. The linear heart tube undergoes looping at E8.5, and during this stage a population of pharyngeal mesoderm-derived cells, the second heart field, contributes to myocardial as well as endocardial components of the right ventricle, the interventricular septum, the venous pole, and the base of the outflow tract (OFT) [1]. After birth, the task of directing oxygenated and deoxygenated blood to the systemic and pulmonary circulatory systems, respectively, is carried out by the OFT. In humans, congenital heart defects occur in almost 1% of newborns and defects in OFT development are some of the most severe heart abnormalities [2]. The OFT consists initially of cells from the heart tube but a major contribution is made later by cells of the anterior heart field and cardiac neural crest cells (cardiac NCC) [3]. Cardiac NCC represent a subpopulation of the cranial neural crest and originate from an area beginning around the middle of the otic placode to the caudal border of somite 3, corresponding to rhombomeres 6–8 [4]. Together these two populations generate a septated ascending aorta and pulmonary trunk during cardiogenesis.

At E10.5 the heart has acquired well-formed chambers, and cardiac NCC migrate to the OFT [5]. Cardiac NCC migrate into the endocardial cushions, which are derived from the heart endocardial layer, and together these two populations form the septum that divides the OFT into systemic and pulmonary outlets. The septum of the OFT starts as a shelf-like structure at the distal end of the OFT. The shelf develops between the fourth aortic arch artery (the future aorta) and sixth aortic arch artery (the future pulmonary trunk), and it expands proximally from the distal end. Cardiac NCC are important for a proper septation process of the OFT and for patterning of the great vessels [6,7]. Cardiac NCC also contribute to the septation process between the left and right ventricles and atria [8] that occurs between E10 and E13 [9]. By E14.5 the heart chambers are completely septated and are connected to the pulmonary trunk and aorta.

Cilia are 1 to 3 μm long protrusions of most cell types in vertebrates, which grow out from a centriole-based basal body located in the cytoplasm, bear a microtubule-based axoneme [10], and are coated by the plasma membrane. Primary cilia possess an intraflagellar transport system (IFT) for proteins and other cargo to travel in and out of the cilium. The IFT proteins act as scaffolds between the protein cargo and the motors kinesin-II and dynein, which are responsible for antero- and retrograde transport, respectively [11]. Primary cilia have recently been revealed as crucial for the development of a number of organs (reviewed in [12]). A clinical correlate of the developmental studies has been the re-definition of a wide variety of human syndromes as ‘ciliopathies’, because the defective genes in question encode proteins that localize to the basal body or the cilium, including Bardet-Biedl, Meckel-Gruber, Alstrom, Joubert, orofaciodigital, and Senior-Loken syndromes, as well as nephronophthisis and polycystic kidney disease (PKD) (reviewed in [13]). In particular, primary cilia are essential for signal transduction of the Hedgehog (Hh) pathway. The three ligands of the Hh family bind to the inhibitory receptor Patched, which, in the absence of Hh ligands, localizes to primary cilia. Upon exposure to ligand, Patched and its ligand move out of the cilium and become internalized within the cytoplasm. In turn, the Hh signal-promoting receptor Smoothened enters into the cilium and executes a proper transduction of Hh signals [14,15].

The recessive mouse mutant *cobblestone* (*cbs*) bears a hypomorphic allele of the gene *Ift88*[16], leading to a reduction of Ift88 mRNA and protein levels in *cbs* embryos by 70% to 80% compared to wild type littermates. Ift88, a component of the IFT system, is essential for the formation and maintenance of primary cilia [17]. *Cobblestone* mutants show a wide range of defects in the central nervous system that are at least partially explained by a deficiency in Sonic hedgehog (Shh) signaling [16]. This prompted us to examine cardiogenesis in this mutant mouse line, as Shh signaling contributes to several important aspects of heart development, including atrioventricular and OFT septation [18-23]. We show that *cbs* embryos display several malformations in cardiac development between E12.5 and E16.5, including interventricular- and interatrial septum defects, persistent truncus arteriosus, arteria lusoria, and pericardial hyperplasia. In this paper, we provide evidence that loss of cardiac cilia correlated with deficiencies in the expression of genes known to be important for OFT and atrioventricular septation, and we present a mechanistic explanation for these phenotypes.

## Methods

### Mouse lines

All experiments were conducted according to the guidelines of the state of Baden-Württemberg, Germany. *Cobblestone* mice were generated as described [16]. Twelve noon of the day of the vaginal plug was assigned the date embryonic day 0.5 (E0.5). For all embryonic stages before E12.5, somite-matched embryos were compared for analysis as follows: E9.5: 21 to 29 somites, E10.5: 35 to 39 somites; E11.5: >45 somites [24]. Genomic DNA was isolated from embryonic and adult tissue as described [25]. For PCR genotyping the following primers were utilized (D14Mit121 F: 5^′^-TTG ACA TCT GGA TAT GAC AAT GC-3^′^; D14Mit121 R: 5^′^-TGT GCA TGT TTG TGT ACA TAT GTG-3^′^; D14Mit259 F: 5^′^-TGG TGT CTC CTT CGG AAT TT −3^′^; D14Mit259 R: 5^′^-TAA ATG TAA AAG GTA AAG GCA ATG G-3^′^) and PCR products were resolved using 12% acrylamide gel electrophoresis.

### Transmission electron microscopy

E12.5 mouse embryos were collected in cold PBS and fixed overnight (O/N) at 4°C with 2.5% glutaraldehyde in 0.1 M PIPES buffer, pH 7.6, containing 2% polyvinylpyrrolidone (MW 25000, Merck, Darmstadt, Germany). Transverse sections (300 μm-thick) of the trunk were prepared using a vibratome (D.S.K. Microslicer DTK-1000, Dosaka EM, Kyoto, Japan). For enhancement of membrane staining, samples were incubated in alkaline diaminobenzidine hydrochloride medium as described previously [26] for 60 minutes, and postfixed with 1.5% osmium tetroxide containing 1.5% potassium ferrocyanide for one hour followed by an additional one hour osmification with 1.5% osmium tetroxide in 0.1 M sodium cacodylate buffer. Finally, the slices were stained *en bloc* in 1% uranyl acetate for 30 minutes, dehydrated through a graded ethanol series and embedded in Epon 812 (Fluka 45345). Series of semithin sections were stained with a modified Richardson methylene blue-azure II solution and used for selection of corresponding areas in wild type hearts. Ultrathin sections were stained with lead citrate and analyzed by electron microscopy using a Zeiss EM 906E.

### Hematoxylin & eosin (H & E) staining

Mouse embryos of different stages (E12.5 to E16.5) were fixed O/N with freshly-prepared 4% paraformaldehyde (PFA) in 0.1 M PBS at room temperature (RT), dehydrated in a graded ethanol series and embedded in paraffin. Serial paraffin sections (510 μm-thick) were treated for 2x 5 minutes with xylol, rehydrated in a descending ethanol series, and washed with distilled H_2_O (dH_2_O). The sections were stained with hematoxylin for 8 to 10 minutes, rinsed briefly in dH_2_O and 15 minutes in tap water. The sections were briefly rinsed in dH_2_O before staining in 0.1% Eosin, rinsed several times in dH_2_O, dehydrated in an ascending ethanol series, treated for 2x 5 minutes with xylol, and embedded in Entellan (Merck).

### Three-dimensional OFT reconstruction

The OpenCAR software ([27]; available from http://opencar.ulster.ac.uk) was used for the three-dimensional reconstruction of the outflow tract of E14.5 old wild type and mutant embryos. The reconstruction was undertaken as previously described [28] with the following modifications. Light micrographs of H & E stained serial sections of the thorax were aligned manually using prominent structures in two adjacent images as landmarks. Subsequently, the individual vasculature was manually traced in each section, which resulted in a set of contours representing each of the components of the outflow tract. The contours were three-dimensionally reconstructed by applying the Delauny method [27], generating renderings that were then exported. Amira 5.3.1 software (Visage Imaging, Richmond, Australia) was used for visualization of the exported three-dimensional reconstructed structures.

**Figure 1:**
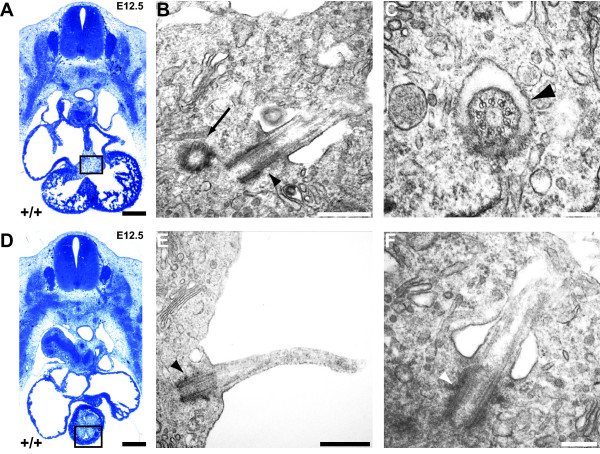
**Primary cilia are present in developmentally**-**important regions of the embryonic heart.** (**A**, **D**) Transverse semithin sections of E12.5 mouse wild type (+/+) embryos showing the areas examined for ciliary ultrastructure in the atrioventricular endocardial cushions (**A**, black box) and the conotruncal endocardial cushions of the truncus arteriosus (**D**, black box). Dorsal is to the top. (**B**, **C**, **E**, **F**) Transmission electron microscopy of primary cilia born by mesenchymal (**B**, **C** ,**F**) or endocardial (**E**) cells of the cushions. Individual cilia are cut transversely (**C**) or longitudinally (**B**, **E** ,**F**). Note the ‘9 + 0’ morphology characteristic of primary cilia (**C**) and the absence of a central microtubule doublet (**B**, **C**, **E**, **F**). (**B**) Black arrow, daughter basal body adjacent to a primary cilium. (**B**, **E**, **F**) Arrowhead, basal body of a primary cilium. (**C**) Black arrowhead, ciliary axoneme. Scale bars: (**A**, **D**) 300 μm, (**B**, **E**) 500 nm, (**C**, **F**) 200 nm. E12.5, embryonic day 12.5.

For the three-dimensional OFT reconstructions shown in Figure 1 and Additional file 2, serial sections were photographed and printed and transferred to balsa wood discs (1:60 magnification). The resulting models were then photographed and sketched with close reference to the photographs and the original micrographs. The vertical axis was artifically exaggerated by a factor of three in order to simplify understanding.

### Immunohistochemical analysis

E10.5 to E12.5 old mouse embryos were collected in cold 0.1 M PBS, and embryonic tail samples were collected separately for DNA extraction and genotyping. The embryos were fixed O/N at 4°C in 4% PFA in 0.1 M PBS. After rinsing in 0.1 M PBS for 2 × 30 minutes the embryos were treated in an ascending sucrose series (10%, 20%, and 30% in 0.1 M PBS) and mounted in optimal cutting temperature (OCT) embedding medium (Leica Microsystems, Wetzlar, Germany). Stainings of 10 μm to 12 μm cryosections were performed as described [29] with the following primary antibodies: anti-acetylated alpha tubulin (clone 6-11B-1, Sigma, Munich, Germany) 1:1000; anti-gamma tubulin (clone GTU-88, Sigma) 1:1000; anti-phosphorylated histone H3 (Ser10, rabbit 06 –570; Millipore Corporation, Schwalbach am Taunus, Germany) 1:200; anti-cleaved caspase-3 (Asp175, #9661, 1:200; Cell Signaling Technology, Danvers, Massachusetts, USA) 1:200; anti-p75 [30] 1:500; anti-AP2α (clone 3B5, Developmental Studies Hybridoma Bank, Iowa City, Iowa, USA) 1:50; anti-α-actinin (clone EA-53, Sigma) 1:2000; anti-Arl13b (kind gift of Tamara Caspary, Atlanta, Georgia, USA) 1:1500; anti-Ift88 (ab42497, goat polyclonal, Abcam plc, Cambridge, England) 1:500; anti-Islet 1 (clone 39.4D5, Developmental Studies Hybridoma Bank) 1:1000. Secondary antibodies were employed as described [29], with the exception that for colabeling experiments to detect cilia, the anti-acetylated alpha tubulin and the anti-gamma tubulin antibodies were detected with isotype-specific anti-mouse immunoglobulin G1 (IgG1) and IgG2b secondary antibodies, respectively. All secondary antibodies were purchased from Invitrogen, Darmstadt, Germany.

### Statistical analysis

**Figure 2:**
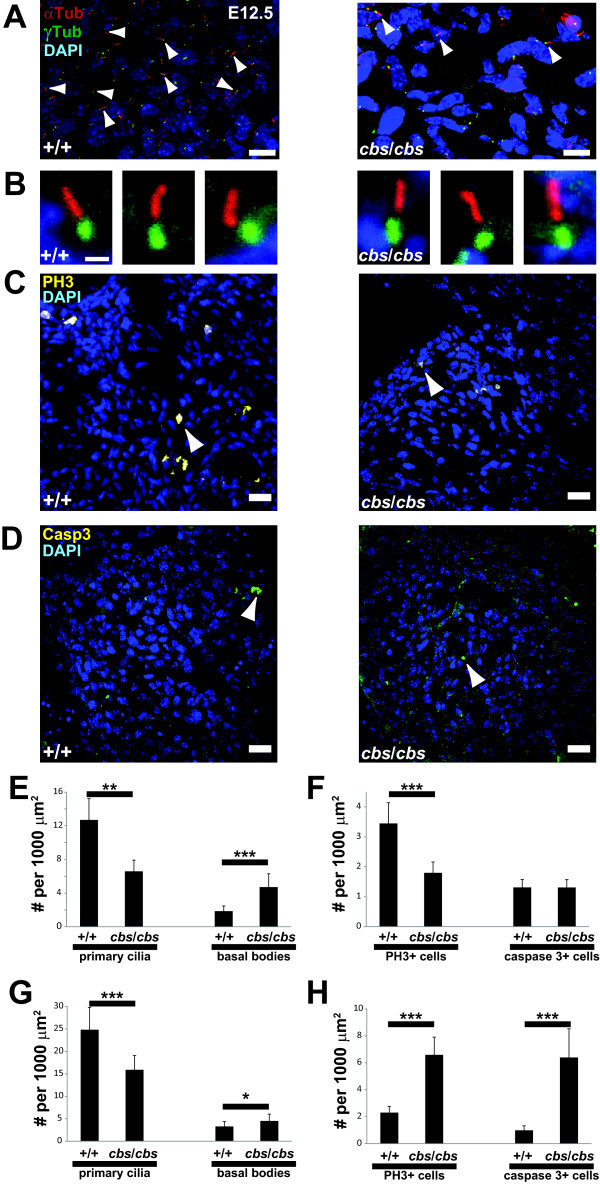
**Primary cilia are reduced in number in the endocardial cushions of *cbs *mutants.** (**A**) Immunofluorescence analysis of primary cilia (arrowheads) in atrioventricular endocardial cushions of E12.5 wild type (+/+) and *cbs/cbs* heart using antibodies recognizing the ciliary axoneme (anti-acetylated α-tubulin, αTub, red) and the basal body (anti-γ-tubulin, γTub, green). (**B**) Individual cilia in E12.5 wild type (+/+) and *cbs/cbs* endocardial cushions, labelled with antibodies recognizing Arl13b (red) to mark the ciliary axoneme and γ-tubulin (green) to mark the basal bodies. (**C**, **D**) Immunofluorescence analysis of proliferation (**C**) and apoptosis (**D**) in endocardial cushions of E12.5 wild type (+/+) and *cbs/cbs* heart using antibodies recognizing phosphorylated histone H3 (C, PΗ3, yellow) and cleaved, activated caspase-3 (**D**, Casp3, green). (**A** to **D**) DAPI-labeled nuclei in blue. Scale bars: **A**, 10 μm; **B**, 1 μm **C**, **D**, 20 μm. (**E** to **H**) Quantification of primary cilia and basal body density (**E**, **G**) as well as proliferation (PH3+ cells) and apoptosis (caspase-3+ cells) (**F**, **H**) in atrioventricular endocardial cushions (**E**, **F**) and pericardium (**G**, **H**), respectively, of E12.5 wild type (+/+) and *cbs/cbs* heart. *P <0.05, **P <0.01, ***P <0.001, Student’s *t* test, n >3 for all comparisons. *cbs*, *cobblestone*; DAPI*,* 4'-6-diamidino-2-phenylindole, E12.5; embryonic day 12.5.

To analyze the data presented in Figure 2, Student's t-test was used, using two tailed, two-sample equal variance settings in Excel (Microsoft Office 2010).

### *In situ* hybridization

*In situ* hybridizations on cryosections were performed as described [31]. *In situ* hybridizations on paraffin sections were performed as described [16]. Probes: *Bmp2* &*Bmp4*[32], *Pitx2c* (kindly provided by Axel Schweickert).

### Whole mount *in situ* hybridization (WISH)

E10.5 mouse embryos were collected in cold 0.1 M PBS, and embryonic tail samples were collected separately for PCR genotyping. The embryos were fixed O/N at 4°C in 4% PFA in 0.1 M PBS. PFA was washed out with 0.1 M PBS, the embryos were dehydrated in an ascending methanol series, kept O/N in 100% methanol at −20°C, and then rehydrated in a descending methanol series. The embryos were washed 2× with 1× PBST (0.1 M PBS; 0.1% Tween-20) for 5 minutes, bleached for 1 hour with 6% H_2_O_2_ in 1× PBST, washed 3× 5 minutes with 1× PBST, treated with proteinase K (10 μg/ml) for 15 minutes, washed once for 10 minutes with 2 mg/ml glycine in 1× PBST, washed 2× 5 minutes with 1× PBST, postfixed for 20 minutes in 4% PFA and 0.2% glutaraldehyde in 1× PBST, and washed 2× 5 minutes in 1× PBST. The embryos were treated for a slow equilibration in the hybridization solution by incubating them 1× 10 minutes in a 1:1 mixture of hybridization solution/1× PBST, 1× 10 minutes in hybridization solution, 1× 60 minutes in hybridization solution at 70°C. The hybridization solution was replaced with fresh hybridization solution together with digoxigenin-labeled antisense-RNA probes and incubated O/N at 70°C. The embryos were washed 3× 30 minutes in prewarmed solution I (50% formamide, 5× SSC, pH 4.5; 1% SDS) at 70°C, 3× 30 minutes in prewarmed solution III (50% formamide; 2× SSC, pH 4.5) at 65°C, 3× 5 minutes in 1× TBST (25 mM Tris, pH 7.5; 150 mM NaCl; 2 mM KCl; 0.1% Tween-20) at RT, and subsequently incubated 1× 2 hours in blocking buffer (10% heat-inactivated sheep serum; 0.1% Boerhinger Mannheim blocking reagent; 1× TBST) at RT. After blocking, the anti-digoxigenin-AP antibody/Fab fragments (Roche, Mannheim, Germany) diluted in fresh blocking buffer were added to the embryos and incubated O/N at 4°C. The embryos were washed 3x 5 minutes and 6× 1 hour in 1× TBST at RT, O/N in 1× TBST at 4°C, 3x 10 minutes in NTMT (100 mM NaCl; 100 mM Tris–HCl, pH 9.5; 50 mM MgCl_2_; 0.1% Tween-20; 2 mM levamisole) at RT, and then developed in the dark in reaction mix (NBT/BCIP stock solution (Roche) diluted 1:50 in NTMT). When the reaction was judged complete, the embryos were washed 1x 10 minutes in PBST, postfixed 10 minutes in 4% PFA at RT, washed 3x 5 minutes in 0.1 M PBS, and stored in 0.1 M PBS at 4°C. For documentation, embryos were cleared in 100% glycerol and photographed with an AZ100 Multizoom microscope (Nikon). Probes: *Shh, Ptch1*[16], *Sox10* (kindly provided by Peter J. Scambler), *Hand1* ([33], kindly provided by Eric Olson), *Nkx2.5* (kindly provided by Thomas Braun), *Gli1* (kindly provided by Sandra Blaess).

## Results

### Primary cilia are present in developmentally-important regions of the embryonic heart

Cilia have been recently reported to be present throughout developing embryonic heart [34], using an immunofluorescent approach. However, an ultrastructural analysis is obligatory to definitively determine whether these cilia are primary or motile. Embryonic wild type mouse heart was examined using transmission electron microscopy (TEM) upon transverse sections of E12.5 embryonic trunk. Cilia, with lengths varying between 1.0 μm to 2.0 μm, frequently occurred in both the atrioventricular and conotruncal endocardial cushions (Figure 1). Cilia in the cushion mesenchyme were often found in ciliary pockets of varying depths (Figures 1B, C, F) and showed a random orientation within the extracellular matrix of the cushion, whereas endocardial cells oriented their primary cilia to the lumen of the inflow and outflow tract, respectively (Figure 1E). Transverse sections revealed a ‘9 + 0’ morphology in the proximal cilium (Figure 1C), and longitudinal sections revealed an absence of a central microtubule doublet (Figures 1B, E, F), confirming them as primary cilia. No cilia bearing a ‘9 + 2’ ultrastructure, indicative of motile cilia, were found in the embryonic heart.

### The *Ift88* hypomorph *cobblestone* displays multiple heart defects

**Figure 3:**
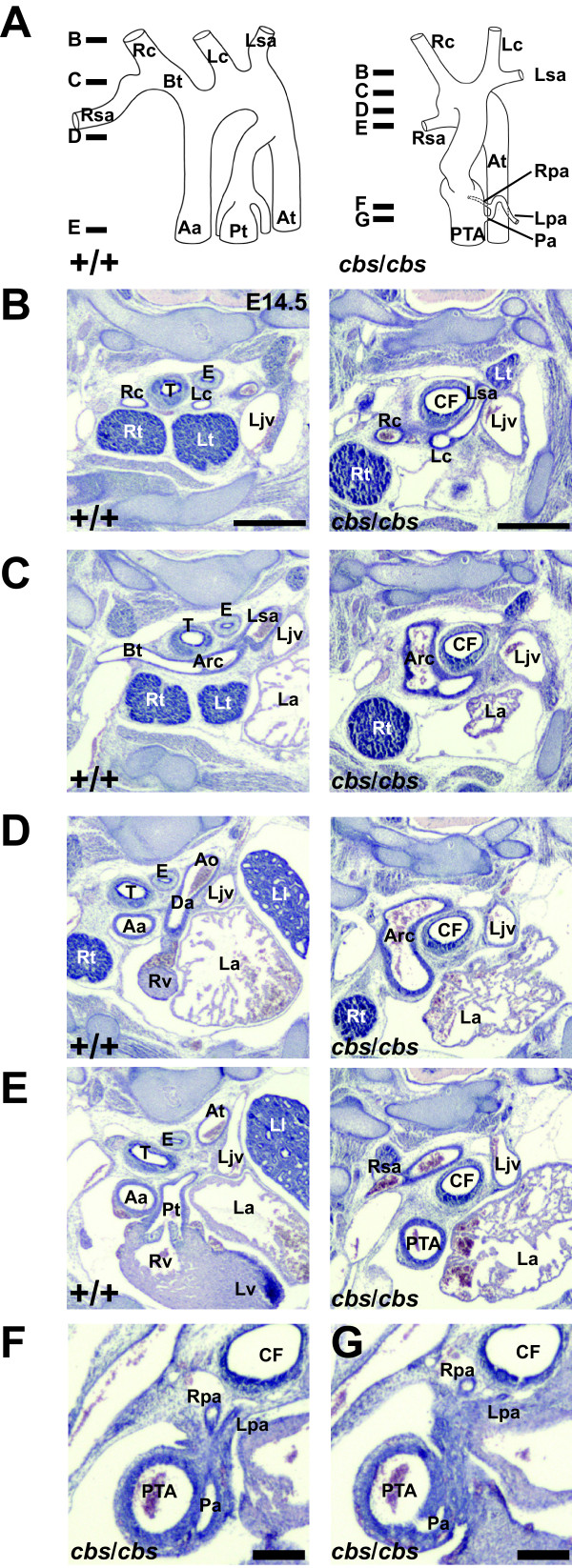
**OFT development is impaired in *cbs *mutants at E14.5.** Serial 10-μm transverse sections of E14.5 wild type (**B**-**E**) and *cbs*/*cbs* (**B**-**G**) embryos were stained with H & E. They are presented from cranial to caudal, and the left side of the body is to the right, dorsal is at the top of each panel. A schematic reconstruction of the OFT is shown (**A**), with lines indicating the planes of the sections (**B**-**G**). In the schematic, the vertical axis has been exaggerated threefold in order to clearly display the complex outflow tract structure. For a description of the defects seen in the *cbs/cbs* mutants, please refer to the results section. Abbreviations (alphabetically arranged): Aa, ascending aorta; Ao, aorta; Arc, aortic arch; At, thoracic aorta; Bt, brachiocephalic trunk; CF, common foregut tube; Da, ductus arteriosus; E, esophagus; La, left atrium; Ljv, left internal jugular vein; Lpa, left pulmonary artery; Lsa, left subclavian artery; Ll, left lung; Lt, left thymus; Lv, left ventricle; Pa, common pulmonary artery; Pt, pulmonary trunk; PTA, persistent truncus arteriosus; Rc, right common carotid artery; Rpa, right pulmonary artery; Rsa, right subclavian artery; Rt, right thymus; Rv, right ventricle; T, trachea. Scale bars: (**B**-**E**) 0.5 mm, (**F**,**G**) 0.2 mm. *cbs*, *cobblestone*; E14.5, embryonic day 14.5; OFT, outflow tract.

**Figure 4:**
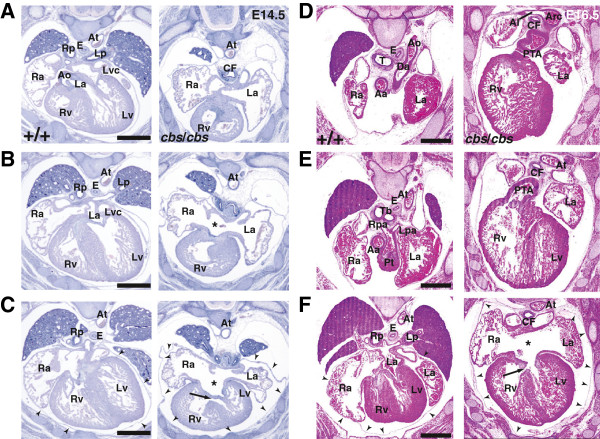
**Atrioventricular septation is impaired in *cbs *mutants.** Serial 10-μm transverse sections of E14.5 (**A**-**C**) and E16.5 (**D**-**F**) wild type and *cbs*/*cbs* embryos were stained with H & E. They are presented from cranial to caudal, and the left side of the body is to the right, dorsal is at the top of each panel. Dextrotransposition of the common trunk arising from the right ventricle (**D**,**E**) combined with both ASD (**B**,**C**,**F**) and VSD-pars membranacea (**C**,**F**) was recorded. The pericardium is also enlarged (**C**,**F**). An example of arteria lusoria (‘Al’) is indicated (**D**). Abbreviations (alphabetically arranged): Aa, ascending aorta; Al, arteria lusoria; Ao, aorta; Arc, aortic arch; At, thoracic aorta; CF, common foregut tube; Da, ductus arteriosus; E, esophagus; La, left atrium; Lp, left principal bronchus; Lpa, left pulmonary artery; Lv, left ventricle; Lvc, left superior vena cava; Pt, pulmonary trunk; PTA, persistent truncus arteriosus; Ra, right atrium; Rp, right principal bronchus; Rpa, right pulmonary artery; Rv, right ventricle; T, trachea; Tb, tracheal bifurcation; *, atrial septum defect (ASD); →, ventricular septum defect (VSD); arrowheads, outline of pericardium. Scale bar: 0.5 mm. *cbs*, *cobblestone*; E14.5, embryonic day 14.5.

Homozygous *cbs* mutant E14.5 (Figures 3, 4A to C) and E16.5 (Figure 4D to E) embryos were analyzed in detail using computer-generated three-dimensional reconstructions (Additional file 1) and serial sections (Figures 3, 4, Additional file 2) of the thorax and compared to their wild type littermates. The following defects were recorded. 1) Persistent truncus arteriosus (PTA) including absence of a ductus arteriosus (Figure 3E to G, Figure 4D, E, n = 4/5). 2) Transposition of the common trunk arising from the right ventricle (Figure 3E, F, Figure 4D, E, n = 4/5). 3) Failure of correct formation of the tracheoesophageal septum (common foregut tube, Figure 3B to G, Figure 4A, D to F, n = 5/5) results in esophageal atresia and a tracheoesophageal fistula at the tracheal bifurcation. 4) Bilateral hypoplasia of the lungs (Figure 4A to C, n = 5/5). 5) The lungs were supplied by a common trunk arising as the first branch of the common truncus arteriosus, giving rise to the right and left pulmonary arteries that descended anterior to the common foregut tube (Figure 3F, G, n = 4/5). 6) Abnormal origin of the right subclavian artery, arising as the fourth branch of the aortic arch and passing posterior to the common foregut tube (Figure 3E and Figure 4D (Arteria lusoria), n = 2/5). 7) Both atrial (Figure 4B, C, F) and ventricular septal defects (VSD, pars membranacea, Figure 4C, F, n = 5/5). 8) Gross enlargement of the pericardial sac (Figure 4C, F, n = 12/12). 9) Hypoplasia of the myocardium, resulting in smaller atria and ventricles (Figure 3A to C, n = 2/5). 10) A single case of a double aortic arch [see Additional file 2]. Cardiovascular defects observed were seen in all examined embryos between E14.5 and E16.5 (for example, AVSD, n = 5). Since 80% of the embryos die by E14.5 (n = 34 litters), and almost all by E16.5 (n = 25 litters), detailed examination of the heart defects at later stages proved impossible.

### Primary cilia are deficient in the developing heart of the *cbs* mutant

Many reports of mouse mutants with defects in ciliary proteins have reported either a loss of (for example, [35]) or morphological abnormalities (for example, [36]) in the cilia. In order to investigate the presence of primary cilia in the developing heart, we labeled the ciliary axoneme with an antibody against acetylated α-tubulin and the basal body with an antibody recognizing γ-tubulin. We observed a substantial loss of primary cilia in both the atrioventricular endocardial cushions (Figure 2A, D, 48.1 ± 10.1% reduction, n = 3, *P* <0.01, Student’s *t* test) as well as the pericardium (Figure 2F, 36.0 ± 12.0% reduction, n = 5, *P* <0.001, Student’s *t* test) of E12.5 heart, expressed as the number of cells with identifiable primary cilia per 1000 μm^2^. To account for differences in cell density between wild type and mutant embryos, DAPI-stained nuclei were counted and found to be identical (endocardial cushions: wild type: 18.7 ± 2.3 cells/1000 μm^2^; *cbs*/*cbs*: 19.5 ± 3.4 cells/1000 μm^2^, n = 10, *P* >0.5). High magnification imaging of the primary cilia remaining in the *cbs* mutant appeared normal (Figure 2B), using antibodies recognizing Arl13b to mark the ciliary axoneme [36] and γ-tubulin to mark the basal bodies. In both the endocardial cushions as well as the pericardium, a reduction in the number of primary cilia was accompanied by an increase in the number of cells bearing basal bodies with no adjacent acetylated α-tubulin-positive ciliary axoneme (Figure 2A, E, G). These findings indicate that the fraction of cells bearing cilia decreased in the population, leading to an increase in the fraction of unciliated cells, with basal bodies but no ciliary axoneme. To see if the loss of cilia was accompanied by a change in proliferation, we employed an antibody that recognizes phosphorylated-histone H3, which becomes phosphorylated specifically during mitosis [37]. We observed a 48.2 ± 10.4% reduction in the number of mitotic cells (Figure 2C, F, n = 3, *P* <0.001, Student’s *t* test) in the endocardial cushions. We did not observe any PH3-positive cells bearing cilia in either wild type or mutant hearts.

To address whether the apoptotic rate was changed we used an antibody recognizing cleaved, activated caspase-3, a processed protein that initiates the proteolytic cascade common to both intrinsic and extrinsic apoptotic pathways [38]. We detected no significant change in the apoptotic rate in *cbs* atrioventricular endocardial cushions, compared to wild type (Figure 2D, F, n = 2, *P* >0.05, Student’s *t* test). Unexpectedly, in the pericardium both mitosis (285.8 ± 57.2%, n = 3, *P* <0.001, Student’s *t* test) and apoptotic counts (642.9 ± 214.3%, n = 2, *P* <0.001, Student’s *t* test) increased dramatically (Figure 2H).

### Migration of cardiac neural crest cells into the developing heart is normal in the *cbs* mutant despite the absence of cilia on the migrating cells

**Figure 5:**
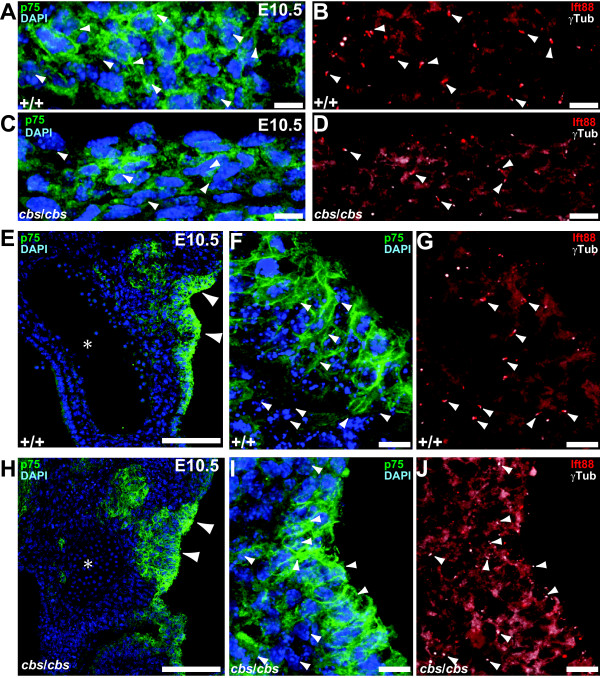
**Cardiac neural crest cells are ciliated during their migration and initial migration is normal in *cbs***/***cbs *mutants.** (**A**-**J**) An antibody recognizing p75 was used to label migratory cardiac NCC in transverse sections of E10.5 wild type (+/+, **A**,**B**,**E**-**G**) and *cbs*/*cbs* (**C**,**D**,**H**-**J**) embryos. (**A**,**B**,**C**,**D**,**F**,**G**,**I**,**J**) In each paired panel (for example, **A**,**B**) a four-channel labeling is split into two panels for ease of interpretation. (**A**,**C**) Initial migration of cohorts of cardiac NCC lateral to the dorsal caudal hindbrain is seen in both wild type and *cbs*/*cbs* embryos. (**B**,**D**) Colabeling of the cells seen in (**A**,**B**) with antibodies recognizing Ift88 (red) to label the ciliary axoneme and anti-γ tubulin (white) to label the basal body demonstrate cilia born by p75-positive cells in both wild type and *cbs*/*cbs* embryos. (**A**-**D**) Arrowheads mark cilia in (**B**,**D**) and are superimposed upon the p75/DAPI stain in (**A**,**C**). Dorsal is to the left, lateral to the top. (**E**,**H**) Arrowheads indicate p75-positive (green) cardiac NCC migrating along the third pharyngeal artery in both wild type and *cbs*/*cbs* embryos. ‘*’: The lumen of the the pharyngeal artery with nucleated red blood cells. (**F**,**G**,**I**,**J**) High magnification view of migrating cardiac NCC in the same area shown in (**E**,**H**). (**G**,**J**) Colabeling of the cells seen in (**F**,**I**) with antibodies recognizing Ift88 (red) to label the ciliary axoneme and anti-γ tubulin (white) to label the basal body demonstrate cilia born by p75-positive cells in wild type embryos but isolated, non-cilium-associated basal bodies in *cbs*/*cbs* embryos. (**F**,**G**,**I**,**J**) Arrowheads mark cilia in (**G**,**J**) and are superimposed upon the p75/DAPI stain in (**F**,**I**). (**E**-**J**) Dorsal is to the top, lateral to the right. (**A**,**C**,**E**,**F**,**H**,**I**) Sections were stained with DAPI to label nuclei. Scale bars: A-D,F,G,I,J, 10 μm; E,H, 100 μm. *cbs*, *cobblestone*; DAPI, 4’-6-diamidino-2-phenylindole; E10.5,embryonic day 10.5; NCC, neural crest cells.

**Figure 6:**
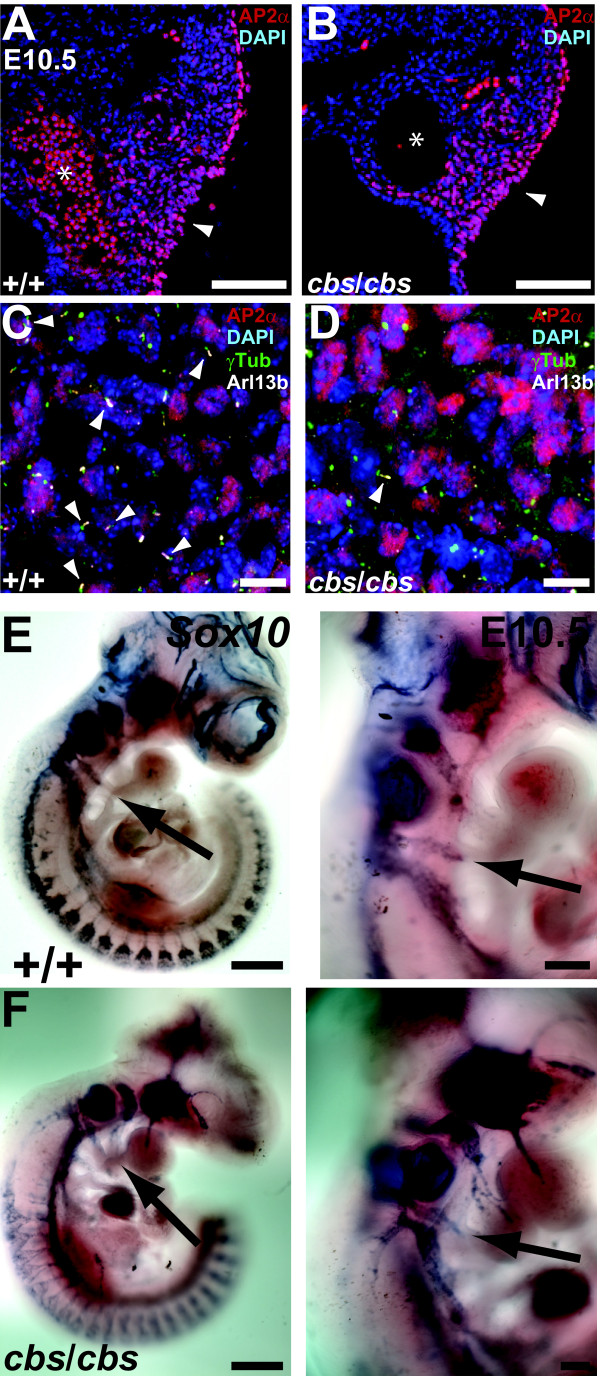
**Cardiac neural crest cells migrate normally into the embryonic *cbs***/***cbs *heart.** (**A**,**B**) Arrowheads indicate AP2α-positive (red) cardiac NCC migrating along the third pharyngeal artery in both wild type (+/+) and *cbs*/*cbs* E10.5 embryos. ‘*’: The lumen of the the pharyngeal artery with nucleated red blood cells (**A**). (**C**,**D**) High magnification view of migrating cardiac NCC in the same area shown in (**A**,**B**). AP2α-positive cells (red) are colabelled with the following markers to demonstrate the presence or absence of cilia: anti-γ tubulin (green) to label the basal body, and anti-Arl13b to label the ciliary axoneme (white). (**C**) Arrowheads indicate primary cilia born by AP2α-positive cells. (**D**) Arrowhead indicates a cilium born by an AP2α-negative cell. (**A**-**D**) Embryos were cut transversely, dorsal is to the top, lateral to the right. Sections were stained with DAPI to label nuclei. (**E**,**F**) *Sox10* WISH on E10.5 wild type (+/+, **E**) and *cbs*/*cbs* (**F**) embryos demonstrate cardiac NCC (arrows) migrating along the third pharyngeal artery into the heart tube in both genotypes. Scale bars: A,B, 100 μm; C,D, 10 μm; E,F, left panels: 500 μm, right panels: 200 μm. *cbs*, *cobblestone*; DAPI, 4’-6-diamidino-2-phenylindole; E10.5,embryonic day 10.5; NCC, neural crest cells; WISH, whole mount *in situ* hybridization.

The cardiovascular phenotypes resulting from cardiac neural crest ablation include PTA, overriding aorta, and double-outlet right ventricle [4]. As we had consistently observed these first two phenotypes in the *cbs* mutants, we decided to investigate the migration pattern of cardiac NCC in *cbs* mutants. First, we co-labeled migrating cardiac NCC with an antibody against the neurotrophin receptor p75 [39] (Figure 5). In both wild type (Figure 5A) and *cbs*/*cbs* (Figure 5C) E10.5 embryos, cardiac NCC migrating from the dorsal caudal hindbrain could be observed clustered lateral to the hindbrain wall. To examine cilia, we labeled the ciliary axoneme with an antibody against Ift88 and the basal body with an antibody recognizing gamma tubulin. p75-positive migrating cardiac NCC in wild type embryos clearly demonstrated cilia (Figure 5A, B). In the *cbs*/*cbs* embryos, migrating cardiac NCC also displayed cilia, although the number of ciliated cells was lower (Figure 5C, D). In the same embryos, cohorts of migrating cardiac NCC could be identified migrating along the third pharyngeal artery toward the heart tube in both wild type (Figure 5E) and *cbs*/*cbs* (Figure 5H) E10.5 embryos. Again, p75-positive migrating cardiac NCC in wild type embryos bore cilia (Figure 5F, G). In contrast, migrating cardiac NCC in *cbs*/*cbs* embryos did not elaborate cilia (Figure 5I, J). In order to use an independent marker for the ciliary axoneme, we employed an antibody recognizing Arl13b [36], colabeling for another marker of migrating cardiac NCC, the transcription factor AP2α [40]. Examination of cells migrating along the third pharyngeal artery of E10.5 embryos revealed results identical to those seen with the anti-p75 antibody (Figure 6A to D). AP2α-positive cells could be found in the *cbs*/*cbs* embryos (Figure 6B), but the cells for the most part did not elaborate cilia (Figure 6D).

Secondly, we employed whole mount *in situ* hybridization (WISH) against mRNA of the transcription factor *Sox10*, which is strongly expressed in migrating neural crest cells [41]. WISH was performed on embryos between E10.5 and E11.5, the period in which cardiac NCC migrate to the heart [42]. A close analysis of the WISH results showed that the cardiac NCC in the *cbs* mutant embryos do in fact migrate from the pharyngeal arches to the heart tube, exhibiting an identical distribution pattern along the pharyngeal arteries (Figure 6E, F).

### Downregulation of Shh signaling in the *cbs* mutant

**Figure 7:**
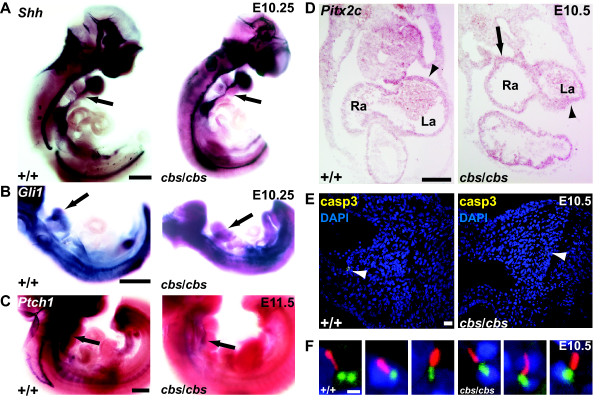
**Shh expression is normal but downstream Shh signaling is compromised in *cbs *mutants.** (**A**-**C**) WISH upon E10.25 (**A**,**B**) and E11.5 (**C**) wild type (+/+) and *cbs*/*cbs* embryos. Arrows indicate *Shh* (**A**), *Gli1* (**B**), and *Ptch1* (**C**) expression in pharyngeal mesoderm adjacent to the heart tube. (**D**) *In situ* hybridization on transverse sections of E10.5 wild type (+/+) and *cbs*/*cbs* embryos, using *Pitx2c* as a probe. Arrowheads indicate expression in the left atrium, while the arrow indicates expression in the right atrium of *cbs*/*cbs* heart. (**E**) Immunofluorescence analysis of apoptosis in pharyngeal arches of E10.5 wild type (+/+) and *cbs/cbs* embryos using an antibody recognizing cleaved, activated caspase-3 (casp3, yellow). Blue: DAPI-labeled nuclei. (**F**) Individual cilia in E12.5 wild type (+/+) and *cbs/cbs* endocardial cushions, labelled with antibodies recognizing Arl13b (red) to mark the ciliary axoneme and γ-tubulin (green) to mark the basal bodies. Traces of DAPI-stained nuclei can be seen in dark blue. Scale bars: A, 400 μm; B,C, 500 μm; D, 200 μm; E, 20 μm; F, 1 μm. *cbs*, *cobblestone*; DAPI, 4’-6-diamidino-2-phenylindole; E10.5,embryonic day 10.5; Shh, Sonic hedgehog; WISH, whole mount *in situ* hybridization.

To date, Shh is the best-documented signal transduction pathway to act through primary cilia [12]. At midgestation and with proximity to the heart tube, Shh is expressed in the ventral spinal cord and notochord, the pharyngeal mesoderm [43], and the pulmonary endoderm [21,44]. Inhibition of Shh signaling from the pharyngeal mesoderm results in severe OFT abnormalities [20]. To examine the effect of the *cbs* mutation upon Shh signaling, we examined *Shh* expression using WISH upon E10.5 embryos. No differences in Shh expression were observed in the embryonic thorax, comparing *cbs* mutants to their wild type littermates (Figure 7A). The observed reduction in the number of primary cilia in the *cbs* mutant (Figure 4) should lead to a reduction in Shh signal transduction. This was indeed the case. Using WISH, we observed a strong reduction in the expression of two downstream targets of Shh signaling, *Gli1* (Figure 7B) and *Ptch1* (Figure 7C), in pharyngeal mesoderm and endoderm.

Shh is also involved in early development in the establishment and maintenance of left/right asymmetry, which takes place initially at the embryonic node [45,46]. A loss of asymmetry can lead to the medical condition *situs inversus*, in which all or some of the internal organs are found on the opposite side of the body. In the mouse, this is manifested by a reversal of heart tube looping, which is found quite often in mutants in ciliary proteins [35,47,48]. The *cbs* mutant never displays reversed heart looping, but when crossed to a full deletion mutant of the *Ift88* it does [16]. Subtler defects have recently been reported in congenital heart defects in humans that can be attributed to ciliary proteins, including OFT abnormalities and atrioventricular septal defects [49,50]. In order to investigate whether subtle laterality defects may occur in the *cbs* mutant, we utilized an *in situ* probe for *Pitx2c*[51,52], a heart-specific isoform of the *Pitx2* gene. *Pitx2* lies directly downstream of left/right determination pathways [53] and is important for atrioventricular development as well as OFT remodeling [54,55]. Examination of E10.5 embryos revealed an atrial expression of *Pitx2c* restricted to the left side of the heart (Figure 7D). In contrast, a bilateral atrial expression of *Pitx2c* was observed in *cbs*/*cbs* hearts (Figure 7D). This phenotype has also been reported in the *Shh* knock-out mouse model [56] and may reflect a left atrial isomerism.

Another reported consequence of a loss of Shh signaling in other mouse mutants is a remarkable increase in apoptotic rates in the pharyngeal mesoderm and endoderm and splanchnic mesoderm [20,22,23,43]. Thus, we examined apoptosis using an antibody recognizing cleaved, activated caspase in E10.5 embryos. Our data revealed very low levels of apoptosis in the pharyngeal mesoderm and endoderm and OFT of both wild type and *cbs* mutant embryos (Figure 7E). Finally, we examined primary cilia in the pharyngeal mesoderm using antibodies recognizing Arl13b to mark the ciliary axoneme [36] and γ-tubulin to mark the basal bodies. As in the atrioventricular endocardial cushions at E12.5 and migratory cardiac NCC at E10.5, we observed a reduction in the fraction of cells bearing cilia in *cbs/cbs* mutants, but the morphology of individual cilia in *cbs/cbs* mutants appeared normal (Figure 7F).

### Defects in cardiac mesoderm differentiation and cardiomyogenesis in the *cbs* mutant

**Figure 8:**
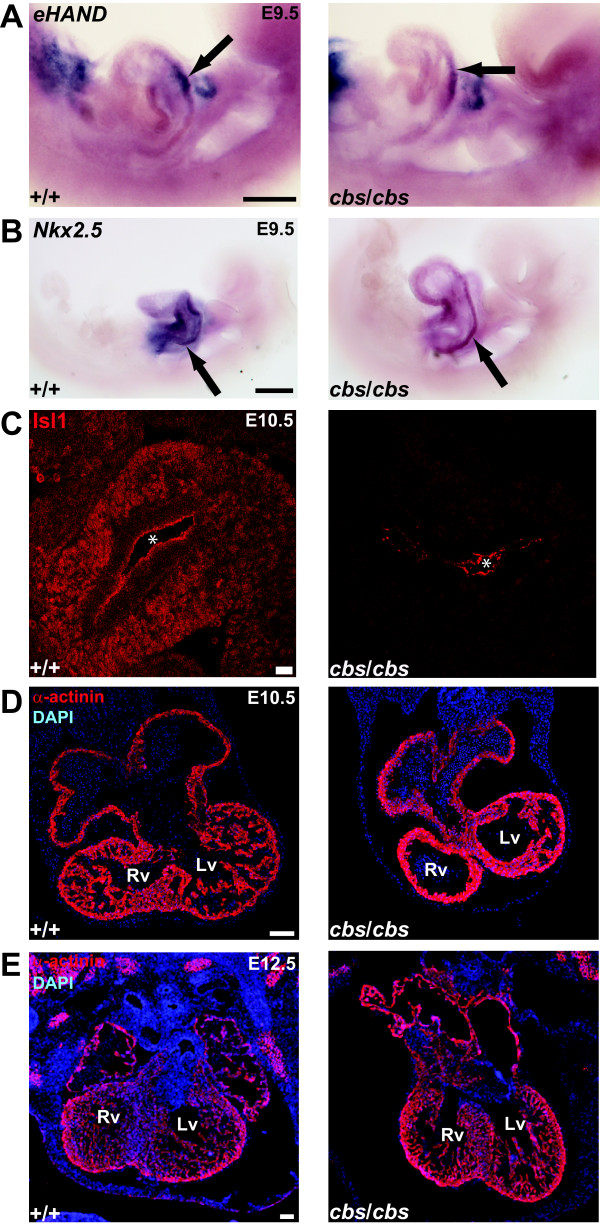
**Analysis of cardiac mesoderm and myocardium formation in *cbs *mutants.** (**A**,**B**) WISH upon E9.5 wild type (+/+) and *cbs/cbs* embryos using probes recognizing *Hand1* (**A**) and *Nkx2.5* (**B**). The left side of the embryo is shown. Ventral is to the top, cranial is to the right. Arrow indicates signal in the left side of the heart tube. (**B**) With *Nkx2.5*, signal is also seen on the right side of the heart tube (data not shown). (**C**) anti-Islet 1 staining (Isl1, red) indicates Islet 1-positive precursors in the splanchnic mesoderm in both wildtype (+/+, c) and *cbs*/*cbs* (d) E10.5 embryos. *: non-specific signal at the apical edge of the common foregut tube epithelia. (**D**,**E**) Transverse sections of (**D**) E10.5 and (**E**) E12.5 wild type (+/+) and *cbs/cbs* embryos stained with an anti-α-actinin antibody (red) to label myocardial heart tissue. Dorsal is to the top, left is to the right. Sections were stained with DAPI to label nuclei. Note the relative lack of trabeculation in the presumptive ventricles (Lv, Rv, presumptive left and right ventricle, respectively) of the *cbs/cbs* heart at E10.5. Scale bars: A,B, 200 μm; C, 20 μm; D,E, 100 μm. *cbs*, *cobblestone*; DAPI, 4’-6-diamidino-2-phenylindole; E9.5,embryonic day 9.5; WISH, whole mount *in situ* hybridization.

To examine differentiation of the first and second heart fields, we employed *in situ* probes against the genes *Hand1*[57] (Figure 8A) and *Nkx2.5*[58] (Figure 8B) and an antibody recognizing Islet1 [59] (Figure 8C), respectively. WISH analysis of E9.5 embryos revealed that *Hand1* expression was found in the pharyngeal arches and restricted to the left side of the heart tube both in wild type and in *cbs/cbs* mutants (Figure 8A). *Nkx2.5* expression was found throughout the heart tube at E9.5 in both wild type and *cbs/cbs* embryos but appeared reduced in expression levels in the *cbs/cbs* mutants (Figure 8B). However, this reduction in expression clearly did not prevent the proper left-sided expression of *Hand1*, in contrast to what is seen in *Nkx2.5* mutant mice [60]. We next examined Islet1 expression, a downstream target of Shh signaling which is downregulated in the spinal cord of ciliary mutants [36,61,62]. More importantly, *Islet1* is known to be crucial for the development of the second heart field and subsequent OFT formation [59]. Immunohistofluorescence with an anti-Islet1 antibody revealed a strong decrease in expression in the splanchnic mesoderm of *cbs* mutant embryos at E10.5 compared to wild type littermates (Figure 8C). To examine cardiomyogenesis, we employed an antibody recognizing the structural protein α-actinin (Figure 8D, E). At E10.5, α-actinin staining revealed a strong decrease in ventricular trabeculation in the *cbs/cbs* embryos (Figure 8D), as has been previously reported for *Ift88*[63] and other ciliary mutants, including *Kif3a* and *Pkd2*[34]. However, at E12.5, trabeculation levels in the ventricle appeared to somewhat recover (Figure 8E).

### Loss of Bmp2/4 expression in both OFT and endocardial cushions of the *cbs* mutant

**Figure 9:**
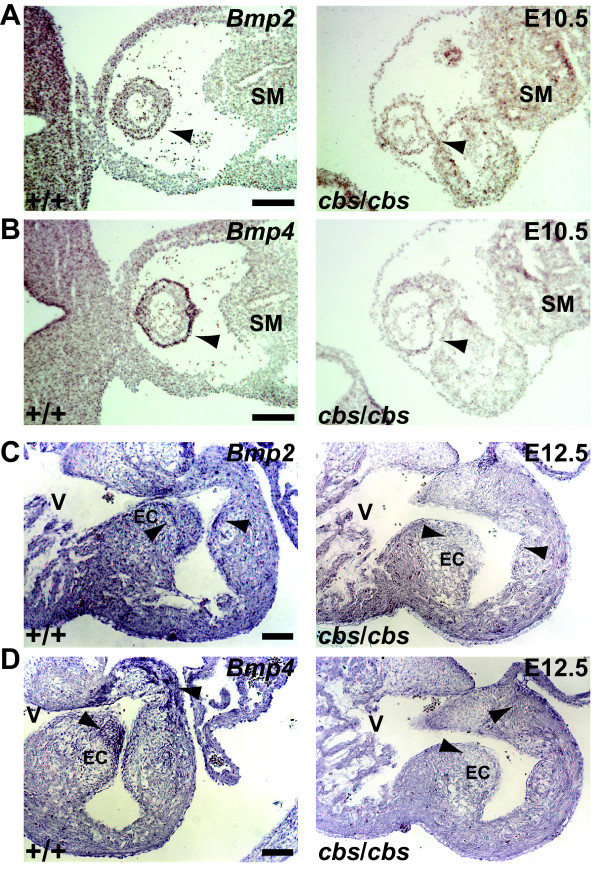
**Loss of *Bmp2 *and *Bmp4 *expression in *cbs *mutant OFT and endocardial cushions.** (**A**,**B**) I*n situ* hybridization of *Bmp2* (**A**,**C**) and *Bmp4* (**B**,**D**) in the OFT (arrow) of E10.5 wild type (+/+) and *cbs/cbs* hearts. For each transverse section, dorsal is to the right. ‘SM’: splanchnic mesoderm. (**C**,**D**) *In situ* hybridization analysis of E12.5 wild type (+/+) and *cbs*/*cbs* embryonic hearts. For each transverse section, dorsal is to the top. ‘V’: right ventricle, EC: endocardial cushion. Arrowheads indicate signals described in text. Scale bars: A,B, 200 μm; C,D, 100 μm. *cbs*, *cobblestone*; E10.5,embryonic day 10.5; OFT, outflow tract.

Elimination of Islet1 expression in the *Isl1* knockout mouse line leads not only to the loss of many second heart field-derived structures, including the OFT, but also to a loss of *Bmp* expression [59]. Therefore, we examined *Bmp2* and *Bmp4* expression in both embryonic OFT, where Bmp signaling is required for normal OFT development [64-69], as well as in atrioventricular endocardial cushions, where *Bmp4* expression is crucial for normal atrioventricular septation [70]. *In situ* analysis of *Bmp2* and *Bmp4* expression in the OFT of E10.5 embryos revealed no change in *Bmp2* expression in the *cbs* mutant (Figure 9A), but a strong reduction in the expression of *Bmp4* (Figure 9B). In contrast, in the endocardial cushions, we observed a complete loss of *Bmp2* (Figure 9C) and *Bmp4* (Figure 9D) expression at E12.5 in the *cbs* mutants.

## Discussion

### Primary cilia and heart development

Defects in heart development in cilia mutants were reported well before cilia were realized to be essential for vertebrate organogenesis (reviewed in [12]). *Pkd2* is a gene that, when mutated, causes autosomal dominant polycystic kidney disease and encodes a transmembrane protein localized to primary cilia. Mice with a targeted mutation in *Pkd2* showed ventricular septum defects and to a lesser extent atrial septum defects [71]. In mice carrying a targeted mutation in *Pkd1,* whose gene product is also localized to primary cilia, atrioventricular septal defects (AVSD), disorganization and thinning of the myocardial wall and double-outlet right ventricles have been observed [72]. A targeted deletion of *Ift88* resulted in a shortening of the distal truncus in the OFT, an apparent hypoplasia of endocardial cushions (ECC), and a reduction in ventricular trabeculation [63], but OFT septation defects and/or AVSD were not examined due to the early death of the embryos. Finally, a targeted deletion of *Kif3a* demonstrated an almost complete lack of ECC and a myocardial hypoplasia at E9.5 [34]. The complete knockouts of *Kif3a* and *Ift88* die at E10.5 and E11.5, respectively, and so an analysis of downstream effects of these deficiencies could not be performed. In addition, several ciliary mutants display an enlarged pericardium, including *Pkd2*[71], *Pkd1*[72], *Kif3a*[73], and *Ift88*[63]. The abundance of gene mutants with similar phenotypes strongly suggests that the observed defects lie with deficiencies in ciliary function *per se* and not with potential non-ciliary roles of the individual proteins. Indeed, a loss of primary cilia in embryonic heart has been reported in the *Kif3a* mutant already at E9.5 [34] and in the *Ift88* mutant at E11.5 [63]. Nevertheless, non-ciliary functions for Ift88 cannot be formally excluded and may contribute to the phenotypes reported here.

Primary cilia are still present in the heart of *Pkd2* mutants at E9.5 [34], yet this mutation displays AVSD later in development. Although the primary cilia were not quantitated in the *Pkd2* mutant, it supports the crucial idea that ciliary function can be disrupted despite the presence of morphologically normal cilia, as seen also in the *cbs* mutant (Figure 2, [16]). The *cbs* mutant is a hypomorph, with expression levels of a normal Ift88 protein at 20% of wild type littermates [16]. The *cbs* mutants survive up to E16.5 because of remaining low levels of *Ift88* expression and reach, therefore, the later stages of cardiogenesis, whereas *Ift88* or *Kif3a* full deletion mutants die before atrioventricular or OFT septation have taken place. Low levels of Ift88 clearly allow for the production of morphologically (Figure 2) and ultrastructurally [16] normal cilia in a subset of cells in the developing embryo. We have previously proposed that these residual numbers of cilia are sufficient to allow earlier developmental processes to take place normally, whereas later embryonic stages are compromised by a progressive loss of cilia [16]. Evidence for this ‘threshold hypothesis’ is provided by the observation that initial left-right determination is completely normal in the *cbs* mutant, but that a cross of the *cbs* allele to the full, targeted deletion of the *Ift88* gene [74] results in a 50% incidence of *situs inversus*[16]. Given that the *cbs* mutants do continue to display cilia in a certain fraction of cells within the developing heart and adjacent pharyngeal arch tissue, we can only speculate as to how this manifests itself on the single-cell level. Given the importance of Ift88 for intraflagellar transport, we find it most likely that even those cells bearing cilia are diminished in their ability to properly transduce Shh signals from the environment. To prove this point, though, we would need to employ a Shh readout that displays single-cell resolution, and this would be a very useful tool for future experiments.

### Primary cilia and the neural crest

Primary cilia and basal bodies have been previously implicated in the migration of NCC. Tobin *et al*. showed that craniofacial defects seen in patients with the ciliopathy Bardet-Biedl syndrome (BBS) could be ascribed to a deficit in the migration of the cranial neural crest to form aspects of the viscerocranium [75]. Examination of a conditional mouse mutant in which NCC were deficient for the ciliary protein Kif3a resulted in hypertelorism and frontonasal dysplasia [76]. As in our study, the authors observed normal migration of craniofacial neural crest but abnormalities after arrival at the migration target, in this case the facial prominences [76]. Although our results clearly show that cardiac NCC migration into the heart tube is taking place in the *cbs* mutants, despite the lack of cilia on migrating cardiac NCCs already within the pharyngeal arches, they do not necessarily contradict the reported role of BBS proteins in neural crest migration [75]. First, the presence or absence of primary cilia was not examined in either of the two aforementioned studies [75,76] during the actual migration process itself, as we have done here. Thus, it is unclear whether cilia are present or absent in migrating NCC in these mutant models. But even if primary cilia are lost in BBS mutants, where direct defects in migration *per se* were demonstrated, it does not necessarily mean that this is a ciliary phenomenon, as the BBS proteins are mostly localized to the basal body and may in any case have non-basal-body functions in this particular population. Second, there may be a difference in the control by primary cilia in neural crest migration from different axial levels. The cranial neural crest that populates the facial prominences arises from a more cranially-located position along the neural tube than cardiac NCC [77]. Also, in the *cbs* mutant we have observed no defects in either the formation or maturation of dorsal root ganglia (Bradford and Tucker, unpublished data), which arise from more caudally-located neural crest than cardiac NCC, suggesting that migration and later development of trunk neural crest is intact. Third, the results linking primary cilia and neural crest migration were reported for zebrafish [75], which may of course additionally reflect species-specific differences in the function of primary cilia. Finally, there may be subtle differences in neural crest migration patterns that we have not detected with our immunohistochemical and WISH analyses.

OFT defects have not been recorded for mutants with myocardial or endocardial-specific knockouts of Bmp2/4 receptors [64,78-80]. This suggests that Bmp2 and Bmp4 produced within the heart are acting upon the migratory cardiac NCC to effect proper OFT formation. Indeed, a neural crest-specific deletion of the BMP receptors Alk3 (*Bmpr1a*) [69] or of Alk2 (*Acvr1*) [66] demonstrated a defective colonization of the heart by cardiac NCC and a concomitant OFT defect. Since we do observe normal migration of cardiac NCC in the *cbs* mutant, this suggests that the heart defects observed lie at least in part in defective signaling of Bmp2/4 to post-migratory cardiac NCC cells. However, endogenous cells of the anterior heart field clearly also play a role in OFT septation, and are also responsive to Shh signaling [20]. Future experiments will further refine the roles of ciliary proteins in heart development with the use of conditional inactivation of ciliary genes using appropriate Cre deleter lines.

### Shh and BMP signaling and OFT formation

Several lines of evidence point to an involvement of Shh in OFT formation. Both the *Shh* deletion mutant [23] as well as chicken embryos treated with the Smoothened inhibitor cyclopamine [18] displayed persistent truncus arteriosus and pulmonary atresia. In the mouse *Shh* mutant, these anomalies could largely be attributed to massive defects in the migration of cardiac NCC, a situation not seen in the *cbs* mutant. Both a conditional mutant in *Smoothened*, in which the gene is deleted in the Isl1-positive population [22], and a *Shh* conditional mutant crossed to the Nkx2.5::CRE deleter strain [20], displayed defects in OFT septation. Interestingly, cyclopamine treatment of embryonic chicken resulted in no defects in cardiac NCC migration but did result in OFT defects, suggesting that Shh signaling is also important for OFT development after the cardiac NCC have established residency in the OFT [18].

Bone morphogenetic proteins (BMPs) play critical roles during OFT formation [81]. *Bmp2* and *Bmp4* are expressed in the second heart field and subsequently in the OFT from E8.5 onwards [67,70,82,83]. A cardiac-specific deletion of *Bmp2* using a Nkx2.5::Cre deleter line revealed no defects in OFT formation [84,85], but a similar experiment using an inducible *Bmp4* mutant resulted in defective OFT septation [67]. A more limited deletion of *Bmp4* from the anterior heart field also resulted in loss of OFT septation, suggesting that the crucial source of Bmp4 lies in anterior heart field-derived myocardium [68].

How do Shh and Bmp2/4 signaling pathways interact? In the chicken, treatment with the Smoothened agonist SAG led to an increase in both *Bmp2* expression as well as downstream Smad1/5/8 signaling in the OFT [86]. Although it is not clear whether Shh is responsible for the initial establishment of *Bmp2/4* expression, we speculate that the loss of primary cilia in the *cbs* heart prevents a proper transduction of Shh signaling and thus causes a failure in promotion/maintenance of *Bmp4* expression, leading to its loss in the OFT with subsequent consequences for septation. Clearly, we observe a reduction in Shh signaling within the pharyngeal arches, as indicated by a reduction in the expression of both *Gli1* (Figure 7B) and *Ptch1* (Figure 7C). The molecule connecting Shh to *Bmp* expression could be the transcription factor Islet1. In the spinal cord, Shh expression is necessary for the induction of Islet1-expressing motor neurons [87], and loss of Shh signal transduction in mouse mutants with defects in primary cilia leads to loss of this neuronal population [36,61,62]. In addition, loss of *Islet1* expression in the heart leads to a downregulation of several Bmp growth factors, including *Bmp2* and *Bmp4*[59], similar to the situation in the *cbs* mutant. The observed loss of primary cilia in the embryonic hearts of the *cbs* mutants could thus prevent the maintenance of a Shh-based signaling pathway that supports Bmp2/4 signaling. In this context, a similar disruption of Bmp signaling in the spinal cord of a mouse mutant for the ciliary protein *Arl13b* has recently been reported [88].

### Shh and BMP signaling and atrioventricular septum formation

As with OFT septum formation, several lines of evidence point to an involvement of Shh in atrioventricular septum formation. Late-term Shh mutants suffer from AVSD [23]. Also, a *Smoothened* conditional mutant crossed to the Isl1::CRE deleter strain displayed AVSD [22]. Shh activation does not seem to occur in the developing heart itself, but in extracardiac progenitor cells that migrate into the heart between E8.0 and E10.5 [19-21]. Deletion of the Smoothened receptor from these atrial septum progenitors [21] resulted in AVSD that looks very similar to the AVSD seen in the *cbs* mutant (Figures 3, 4).

In the mouse, both *Bmp2* and *Bmp4* are expressed in the myocardium of the atrioventricular canal [82,83]. A myocardium-specific deletion of a floxed *Bmp4* allele under control of a cardiac Troponin T (TnT::Cre) deleter line showed that *Bmp4* is essential for proper AV septation after ECC formation [70]. Interestingly, *Bmp4* was not necessary for the formation of the ECC. At E12.5 in the *cbs* mutant, the formation and size of the ECC looks normal, but *Bmp4* expression in the overlying myocardium is completely absent (Figure 8B, C). Our results clearly suggest that one basis for the AVSD in the *cbs* mutant mouse may be the absence of *Bmp4* in the AV ECCs. Taken together, our results potentially identify a novel link between Shh signaling at the primary cilium and Bmp-dependent effects upon cardiogenesis.

## Conclusions

In conclusion, we show for the first time that the ciliary protein Ift88 is essential for the permanent establishment of cardiac cilia, and that reduced expression levels of this protein result in congenital heart abnormalities in later embryogenesis, including OFT and AVSD. In consideration of the high incidence of AVSD in human congenital heart defects, the relatively small fraction of cases with a clear genetic cause, and the high variability in both penetrance and expressivity of these cases and in corresponding animal models [89], our data point to a potentially important linkage to genes of the IFT machinery or basal body of the cilia.

## Note added in proof

Primary cilia have previously been reported in the embryonic ventricle and pericardium of the chick and the mouse [90].

## Abbreviations

ASD: Atrial septum defect; AVSD: Atrioventricular septum defect; BBS: Bardet-Biedl syndrome; Bmp2: Bone morphogenetic protein 2; Bmp4: Bone morphogenetic protein 4; cardiac NCC: Cardiac neural crest cells; *cbs*: *cobblestone*; DAPI: 4’-2-diaminidino-2-phenylindole; DH_2_0: Distilled water; E: Embryonic day; ECC: Endocardial cushions; H & E: Hematoxylin and eosin; Hh: Hedgehog; IFT: Intraflagellar transport system; O/N: Overnight; OFT: Outflow tract; PBS: Phosphate-buffered saline; PCR: Polymerase chain reaction; PFA: Paraformaldehyde; PKD: Polycystic kidney disease; PTA: Persistent truncus arteriosus; RT: Room temperature; Shh: Sonic hedgehog; TEM: Transmission electron microscopy; VSD: Ventricular septum defect; WISH: Whole mount *in situ* hybridization.

## Competing interests

The authors declare that they have no competing interests.

## Authors’ contributions

MAW conducted the experiments and analyzed the data. HARG and KG analyzed the data and criticized the manuscript. KLT conceived of the study, devised and conducted the experiments, analyzed the data, and wrote the manuscript. All authors read and approved the final manuscript.

## Authors’ information

KLT and KG are experienced anatomists in the Institute of Anatomy at the University of Heidelberg and together have been performing ultrastructural and molecular analysis of mutants in ciliary function for the past nine years. MW is a graduate student in the laboratory of KLT, who has spent the past six years investigating the *cbs* mutation. HARG is a pathologist who has been consulting with KLT for 20 years on all matters of anatomy and mouse knockout analysis.

## Supplementary Material

Additional file 1 **Three-dimensional reconstruction of the outflow tract in wild type and *cbs*/*cbs *mutants at E14.5.** A schematic reconstruction of the OFT is shown, generated with the computer programs OpenCAR and Amira from serial sections of E14.5 thorax from wild type (+/+, A,C,E,G) and *cbs*/*cbs* (B,D,F,H) embryos. Views are shown from dorsal-cranial facing downward (A,B), from ventral facing dorsal (C,D), from lateral left (E,F), and from lateral right (G,H). The wild type is colored with arterial vessels in red, pulmonary trunk in blue, and the thoracic aorta in purple. The mutant reconstruction is colored in purple, reflecting the single outlet in the OFT. Abbreviations (alphabetically arranged): Aa, ascending aorta; Arc, aortic arch; At, thoracic aorta; Bt, brachiocephalic trunk; Da, ductus arteriosus; Lc, left common carotid artery; Lpa, left pulmonary artery; Lsa, left subclavian artery; Pt, pulmonary trunk; PTA, persistent truncus arteriosus; Rc, right common carotid artery; Rpa, right pulmonary artery; Rsa, right subclavian artery. Click here for file

Additional file 2 **A case of a double aortic arch in a *cbs*/*cbs *mutant at E14.5.** Serial 10-μm transverse sections of E14.5 wild type (B-E) and *cbs*/*cbs* (G-P) embryos were stained with hematoxylin and eosin. They are presented from cranial to caudal, and the left side of the body is to the right, dorsal is at the top of each panel. A schematic reconstruction of the OFT is shown (A,F), with lines indicating the planes of the sections (B-E,G-P). In the schematic, the vertical axis has been exaggerated threefold in order to clearly display the complex outflow tract structure. Abbreviations (alphabetically arranged): Aa, ascending aorta; Ao, aorta; Arc, aortic arch; At, thoracic aorta; Bt, brachiocephalic trunk; CF, common foregut tube; Da, ductus arteriosus; E, esophagus; La, left atrium; Larc, left aortic arch; Lc, left common carotid artery; Ljv, left internal jugular vein; Lpa, left pulmonary artery; Lsa, left subclavian artery; Ll, left lung; Lt, left thymus; Lv, left ventricle; Pa, common pulmonary artery; Pt, pulmonary trunk; PTA, persistent truncus arteriosus; Rarc, right aortic arch; Rc, right common carotid artery; Rpa, right pulmonary artery; Rsa, right subclavian artery; Rt, right thymus; Rv, right ventricle; T, trachea. Scale bar: (B-E,G-P) 0.5 mm. Click here for file
